# Re-annotation for hypothetical protein CA803_03125 of Methicillin-Resistant Staphylococcus aureus strain SO-1977 isolated from Sudan

**DOI:** 10.6026/97320630015160

**Published:** 2019-03-15

**Authors:** Sofia B Mohamed, Mohamed M Hassan, KA Abdalla Munir, Nusiba I Abdalla, Talal A Adlan, Aisha K Babiker

**Affiliations:** 1 Bioinformatics and Biostatistics Department, National University Research Institute, National University, Sudan

**Keywords:** Hypothetical Protein, MRSA SO-1977 strain, Bioinformatics tool, ADP-ribose hydrolase, Sudan

## Abstract

This study aims to describe the global detection and functional inference of hypothetical protein CA803_03125 from Staphylococcus aureus SO-1977. Computational methods were utilized to study this protein based on sequence similarity and presence of known protein domains. The BLASTp result revealed a significant similarity between the hypothetical protein (CA803_03125) and ADP-ribose hydrolase protein from four S. aureus strains (MW2, MRSA252, COL, and N315). Evolutionary tree diagram revealed a close relationship between the hypothetical protein and proteins of MW2 and COL strains. The physicochemical characterization revealed that all proteins were found to be stable, soluble, hydrophilic and acidic in their nature. The Macro domain was found to exist within all proteins. Moreover, the proteins were of pronounced similarity in terms of primary, secondary and tertiary organization. The protein CA803_03125 (SO-1977) is already known and well characterized as ADP-ribose hydrolase; therefore, we would recommend that its NCBI data has to be updated to be submitted under the name of ADP-ribose hydrolase.

## Background

 Methicillin-resistant Staphylococcus aureus (MRSA) is any strain of a bacterium, Staphylococcus aureus that has developed resistance to most of the available antibiotics. In the last decades, epidemiological studies showed sound increasing of endemic and epidemic spread while its control has become an important concern worldwide [1]. In Sudan, studies have also shown high MRSA incidence rate in hospitals [2]. During recent years, hundreds of bacterial genomes are available, while their annotation is of interest [3]. However, many of these protein functions are still unknown. For this reason, there is an increasing demand for the annotation of the functions of uncharacterized proteins, called "hypothetical proteins" [4]. Hypothetical Protein (HP) is a protein that predicted to be expressed from an open reading frame, but for which there is no experimental evidence of translation [5]. About half of the proteins in most genomes are candidates for HPs. This group is of utmost importance to complete genomic and proteomic information. Detection of new HPs not only offers a presentation of new structures but also new functions [6]. Many protein domains have unknown functions; however, these domains participate in the metabolic pathways of organisms and can cause adverse effects. Several approaches have been developed by scientists with the aid of various computational tools to predict protein function. This has been achieved from information derived from sequence similarity, phylogenetic analysis, conserved domains, motifs and 3D structure [7]. In this study, an extensive insilico analysis was carried out to explain the functional properties of the hypothetical protein CA803_03125 (accession number: OXL90457), of Methicillin- Resistant S. aureus strain SO-1977 using available protein structural and functional analysis tools.

## Methodology

### The hypothetical protein targeted, MSA, and Phylogenetic tree construction

Hypothetical protein CA803_03125 was extracted from our project: whole genome sequencing of MRSA SO-1977 strain, with the GenBank accession number: NFZY00000000. Searches for similar protein sequences were carried out by using protein-Basic Local Alignment Search Tool (BLASTp) [8], with the default algorithm search parameters against the UniProtKB/Swiss-Prot database. Multiple sequence alignment (MSA) of the hypothetical protein from the SO-1977 strain with different strains was performed by Clustal Omega alignment program (https://www.ebi.ac.uk/Tools/msa/clustalo/). Multiple sequence alignments (MSA) result was shaded by BoxShade server [9]. By the same token, the phylogenetic tree of all compared sequences was constructed using MEGA software version.6 [10]. 

### Physicochemical parameters

Computations of various physical and chemical parameters for all proteins were predicted using the ProtParam server. ProtParam was used to determine the following parameters; molecular weight (M. Wt), isoelectric point (pI), amino acid composition, charge (positive or negative), atomic composition, extinction coefficient (EC), estimated half-life, instability index (II), aliphatic index (AI) and grand average of hydropathicity (GRAVY). The amino acids and atomic compositions are self-explanatory [11]. 

### Prediction of functional sites:

Screening for domain sites of the target (hypothetical) plus compared proteins sequences were predicted using the Pfam (protein families) database [12]. Thereafter, transmembrane domains were predicted by using the SOSUI server [13], which distinguishes between the membrane and soluble proteins from amino acid sequences, and predicts the transmembrane helices for the former. 

### Secondary structure prediction:

The secondary structures of proteins were estimated by SOPMA [14].

## Results and discussion:

**Figure 1:**
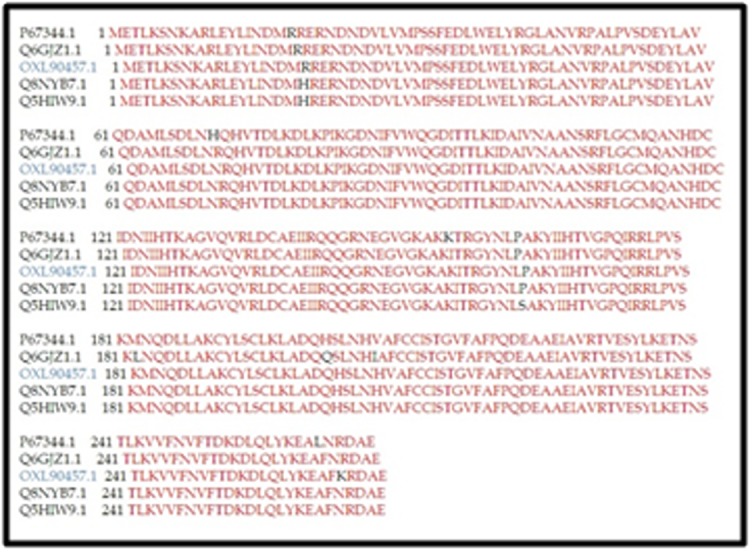
Multiple sequence alignment of the hypothetical proteins from MRSA SO-1977 (labelled blue) with ADP-ribose hydrolase from different S. aureus strains, red regions indicates similar residues. Black regions show nonsimilar.

The protein-BLAST search revealed that the HP CA803_03125 was similar to ADP-ribose hydrolase proteins belong to the other S. aureus spp (Table 1 and Table 2). Furthermore, a small number of variations was detected within an MSA result (Figure 1). The phylogenetic tree showed that protein sequences with accession numbers: 

## Homology Modelling and model validation:

**Figure 2:**
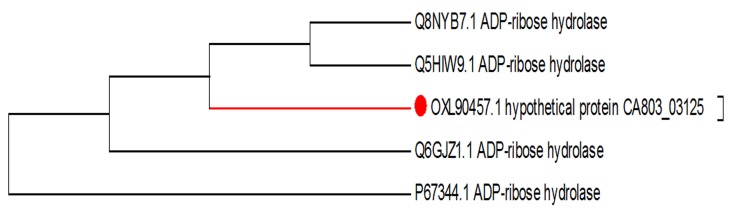
Phylogenetic tree of amino acid sequences of the target hypothetical protein with the ADP-ribose hydrolase of different strains of S. aureus.

**Figure 3:**
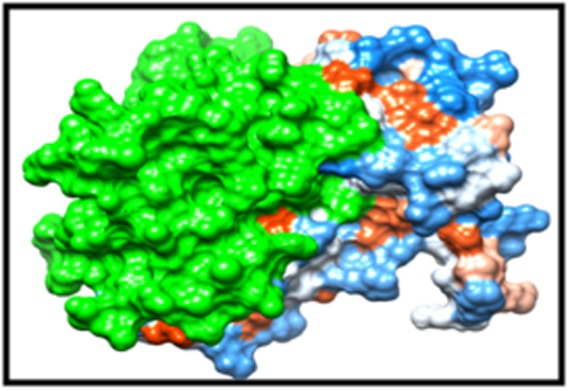
3D structure of the protein model, the green colour shows the MACRO domain

**Figure 4:**
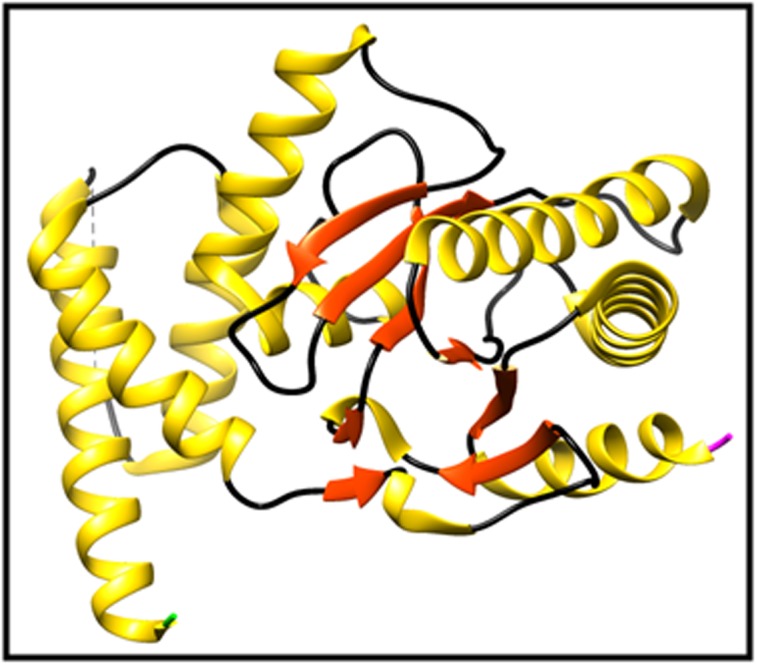
3D structure of the template protein (PDB ID: 5kiv). Golden color shows alpha helices, red color shows extended strand, black color shows Random coil and Beta turns, green color represents the N terminal and pink color represents the C terminal.

The 3D models of the proteins were constructed using Swiss-Model server[15]. Molecular graphics and analyses were performed with the UCSF Chimera package [16].Q8NYB7.1 and Q5HIW9.1 were the closest strains (NCBI Taxonomy IDs: 196620 and 93062 respectively) to HP CA803_03125 (Figure 2). According to physicochemical parameters result, the HP CA803_03125 was found to share same physicochemical properties with the ADP-ribose hydrolases. All the proteins seem to be mildly acidic. The values of instability index for all strains were lower than 40 indicating that all the proteins are stable [17]. All strains showed higher aliphatic indices, which suggested that proteins are stable over a wide temperature range. The GRAVY value is negative, which indicates the hydrophilic and the soluble nature of the proteins [18]. Various parameters were arranged in (Table 3 and Table 4). Pfam search resulted in identifying a MACRO domain between the HP CA803_03125 and ADP-ribose hydrolase proteins from other strains (Table 5). Macro domains are ancient, highly evolutionarily conserved domains that are widely distributed throughout all kingdoms of life. The 'macro fold' is roughly 25kDa in size and is composed of a mixed a-β fold with similarity to the P loop-containing nucleotide triphosphate hydrolases. They function as binding modules for metabolites of NAD+, including poly (ADPribose) (PAR), which is synthesized by PAR polymerases (PARPs) (Figure 3 and Figure 4) [19]. By the same token, all proteins were classified as soluble proteins and the hydrophobicity range was found from - 0.254753 to -0.292857. The secondary structure information may give insights into the higher order structure and functional annotation of the protein [20]. The secondary structure of HP CA803_03125 and ADP-ribose hydrolase proteins was found to be the same. The a-helices were found dominant, followed by random coils, extended strands (β-sheets) and β-turns in all proteins (Table 6). Furthermore, homology modeling for all proteins was built based on a single model template (PDB ID 5kiv) which was the most similar to all of them with varying similarity ranging from 97.74 - 100%. In addition, the GMQE (Global Model Quality Estimation) scores were found to be 0.97 in all proteins, which are very close to 1, as higher number indicates higher reliability (Table 7).

## Conclusion

In this study, the hypothetical protein (OXL90457) from SO-1977 was predicted and identified to be ADP-ribose hydrolase protein. The bacterial MACRO domains are known to influence processes that are crucial for the survival and virulence of bacteria in the host environment. Therefore, MACRO domain would be a subject for further investigations in order to understand the host-pathogen interactions and to explore novel therapeutic routes. The hypothetical protein (OXL90457) should be updated in the NCBI database to be included under the name of ADP-ribose hydrolase.

## Conflict of Interest

Authors declare no conflict of interest

## Figures and Tables

**Table 1:** The Most significance of the highest similarity sequences (BLAST result)

Protein	Strain	Accession number	Identity %
ADP-ribose hydrolase	S. aureus subsp. aureus MW2	Q8NYB7.1	98%
ADP-ribose hydrolase	S. aureus subsp. aureus MRSA252	Q6GJZ1.1	98%
ADP-ribose hydrolase	S. aureus subsp. aureus COL	Q5HIW9.1	98%
ADP-ribose hydrolase	S. aureus subsp. aureus N315	P67344.1	98%

**Table 2:** Percentage of each amino acid composition

Amino Acid	HP (OXL90457)	Q8NYB7	Q6GJZ1	Q5HIW9	P67344
Ala (A)	8.30%	8.30%	8.30%	8.30%	8.30%
Arg (R)	6.00%	5.60%	6.00%	5.60%	5.60%
Asn (N)	6.40%	6.80%	6.80%	6.80%	6.80%
Asp (D)	7.90%	7.90%	7.90%	7.90%	7.90%
Cys (C)	2.60%	2.60%	2.60%	2.60%	2.60%
Gln (Q)	4.50%	4.50%	4.90%	4.50%	4.50%
Glu (E)	5.30%	5.30%	5.30%	5.30%	5.30%
Gly (G)	4.10%	4.10%	4.10%	4.10%	4.10%
His (H)	2.30%	2.60%	1.90%	2.60%	2.60%
Ile (I)	6.40%	6.40%	6.80%	6.40%	6.00%
Leu (L)	10.50%	10.50%	10.90%	10.50%	10.90%
Lys (K)	6.80%	6.40%	6.40%	6.40%	6.80%
Met (M)	2.30%	2.30%	1.90%	2.30%	2.30%
Phe (F)	3.40%	3.40%	3.40%	3.40%	3.00%
Pro (P)	3.00%	3.00%	3.00%	2.60%	3.00%
Ser (S)	4.50%	4.50%	4.50%	4.90%	4.50%
Thr (T)	4.50%	4.50%	4.50%	4.50%	4.50%
Trp (W)	0.80%	0.80%	0.80%	0.80%	0.80%
Tyr (Y)	3.00%	3.00%	3.00%	3.00%	3.00%
Val (V)	7.50%	7.50%	7.10%	7.50%	7.50%
Pyl (O)	0.00%	0.00%	0.00%	0.00%	0.00%
Sec (U)	0.00%	0.00%	0.00%	0.00%	0.00%

**Table 3:** Atomic composition of HP (OXL90457) and similar proteins

Atomic composition	OXL90457	Q8NYB7	Q6GJZ1	Q5HIW9	P67344
Carbon C	1330	1328	1329	1326	1325
Hydrogen H	2133	2122	2132	2120	2125
Nitrogen N	375	374	374	374	375
Oxygen O	398	399	400	400	399
Sulfur S	13	13	12	13	13
Formula	C1330H2133N375O398S13	C1328H2122N374O399S13	C1329H2132N374O400S12	C1326H2120N374O400S13	C1325H2125N375O399S13
Total number of atoms	4249	4236	4247	4233	4237

**Table 4:** Various physicochemical properties of hypothetical (OXL90457.1) and ADP-ribose hydrolase proteins

Protein ID	Molecular weight	Positive charge	Negative charge	Extinction coefficient	GRAVY	Theoretical isoelectric point	Instability index	Aliphatic index	Estimated half life
OXL90457	30161.62	34	35	22920	-0.271	6.6	35.68	96.05	>10 hours
Q8NYB7	30128.5	32	35	22920	-0.265	6.18	35.3	96.05	>10 hours
Q6GJZ1	30134.53	33	35	22920	-0.263	6.23	37.5	97.89	>10 hours
Q5HIW9	30118.46	32	35	22920	-0.262	6.18	33.85	96.05	>10 hours
P67344	30109.5	33	35	22920	-0.293	6.39	34.61	96.05	>10 hours

**Table 5:** Domains identified and their positions for all protein sequences

Protein	Domain	Position
OXL90457	MACRO	103-277
Q8NYB7	MACRO	103-277
Q6GJZ1	MACRO	103-277
Q5HIW9	MACRO	103-277
P67344	MACRO	103-277

**Table 6:** The percentages of secondary structure prediction

Protein ID	Alpha helix %	Extended strand %	Random coil %	Beta turn %
OXL90457	47.91	15.22	28.52	8.37
Q8NYB7	47.37	15.04	27.82	9.77
Q6GJZ1	47.37	15.04	28.95	8.65
Q5HIW9	47.74	15.04	27.44	9.77
P67344	46.62	14.29	29.7	9.4

**Table 7:** Homology modelling and models validation

Protein ID	PDB ID	Identity %	GMQE score
OXL90457	5kiv	98.5	0.97
Q8NYB7	5kiv	98.5	0.97
Q6GJZ1	5kiv	98.12	0.97
Q5HIW9	5kiv	97.74	0.97
P67344	5kiv	100	0.97
